# CRISPR/Cas9: Principle, Applications, and Delivery through Extracellular Vesicles

**DOI:** 10.3390/ijms22116072

**Published:** 2021-06-04

**Authors:** Katarzyna Horodecka, Markus Düchler

**Affiliations:** Department of Bioorganic Chemistry, Centre of Molecular and Macromolecular Studies, Polish Academy of Sciences, 112 Sienkiewicza Street, 90-363 Lodz, Poland; horodeckak@gmail.com

**Keywords:** CRISPR/Cas9, extracellular vesicles, exosomes, microvesicles

## Abstract

The establishment of CRISPR/Cas9 (clustered regularly interspaced short palindromic repeats/CRISPR-associated protein 9) technology for eukaryotic gene editing opened up new avenues not only for the analysis of gene function but also for therapeutic interventions. While the original methodology allowed for targeted gene disruption, recent technological advancements yielded a rich assortment of tools to modify genes and gene expression in various ways. Currently, clinical applications of this technology fell short of expectations mainly due to problems with the efficient and safe delivery of CRISPR/Cas9 components to living organisms. The targeted in vivo delivery of therapeutic nucleic acids and proteins remain technically challenging and further limitations emerge, for instance, by unwanted off-target effects, immune reactions, toxicity, or rapid degradation of the transfer vehicles. One approach that might overcome many of these limitations employs extracellular vesicles as intercellular delivery devices. In this review, we first introduce the CRISPR/Cas9 system and its latest advancements, outline major applications, and summarize the current state of the art technology using exosomes or microvesicles for transporting CRISPR/Cas9 constituents into eukaryotic cells.

## 1. The CRISPR/Cas9 System for Targeted Genetic and Epigenetic Manipulations

The discovery of CRISPR was one of the most revolutionary breakthroughs in science and, as many other priceless findings, was brought to light by coincidence. The first sequences currently known as CRISPR were discovered in E. coli K12 over 30 years ago. Ishino et al. observed five homologous 29 bp (base-pair) repeats spaced by 32 bp sequences in the 3′-end flanking region of the *iap* (alkaline phosphatase) gene coding for a protein responsible for the isozyme conversion of alkaline phosphatase [1]. At the end of the 20th century, a Spanish scientist, Francisco Mojica, began extensive studies of repeated sequences separated by spacers, which originated from his examination of *Haloferax mediterranei*, an extremely halophilic halobacterium [2]. Similar 30 bp long repeats were later found in other halophilic organisms [3], however, their function was thought to be associated with replicon partitioning. Bioinformatics analyses revealed numerous other species including pathogenic bacteria such as Escherichia coli, Salmonella typhi, or Mycobacterium tuberculosis that contained clusters of repeated elements referred to as Short Regularly Spaced Repeats (SRSRs) [4], which were later renamed as Clustered Regularly Interspaced Short Palindromic Repeats (CRISPR) [5].

What renders CRISPR different from other repetitive DNA sequences is that the repeats (from 21 bp to 37 bp long) are interspaced by similarly sized non-repetitive DNA and they are clustered in one or several loci on the chromosome. The CRISPR motifs were found in over 40 archaea and bacteria at that time, but were absent in viral or eukaryotic genomes [5]. To date, ~36% of bacteria and ~75% of archaea were found to contain the CRISPR-Cas system [6]. The origin and function of these mysterious recurrent motifs were unknown until 2005 when sequence similarities with foreign genetic elements including bacteriophages and plasmids were discovered by three independent groups. Moreover, prokaryotes containing CRISPRs were resistant to viruses or plasmids containing sequences matching the spacers. This led to the assumption that CRISPR plays a role in providing immunity against foreign DNA [7,8,9]. The hypothesis was confirmed while studying Streptococcus thermophilus, which is commonly used in the dairy industry. Barrangou et al. [10] observed that strains that were resistant to bacteriophage infection contained phage-derived sequences representing the spacers at CRISPR loci. Interestingly, the resistance was not acquired when single nucleotide polymorphisms were observed between the spacer and the phage sequence and so exactly matching sequences were essential for gaining immunity. Moreover, inactivation of *cas5* (currently known as *cas9*) and *cas7* genes lead to distinguishing the roles of cas7 in the synthesis of repeats and spacers and cas5 (cas9) in acquired immunity.

Further studies revealed the key elements of CRISPR machinery, which relies on the Cas9 enzyme with a nuclease activity that is guided by short CRISPR RNAs (crRNAs) transcribed from the spacer sequences [11,12]. The missing piece of the puzzle was uncovered in 2011 when trans-encoded small RNA (tracrRNA) was shown to be required for maturating crRNAs [13] and the later activating crRNA-guided DNA cleavage [14]. After the essential components of the CRISPR system, namely Cas9, crRNA, and tracrRNA, were recognized, it turned out that a single chimeric RNA complementary to the target sequence could mimic the tracrRNA:crRNA duplex and act as a single guide RNA (sgRNA) for Cas9 [14].

The possibility of programming Cas9 to theoretically target any DNA sequence and generate cleavage led to the idea of utilizing the CRISPR system for genome editing. An endogenous feature that bacteria and archaea both own to acquire immunity was soon incorporated into a mammalian model. In 2013, human embryonic kidney (HEK) 293 FT cells were transfected with SpCas9 (*Streptococcus pyogenes* Cas9) and a guide RNA targeting *EMX1* (Empty Spiracles Homeobox 1) or *PVALB* (Parvalbumin) loci, which generated site-specific double strand breaks. The DSBs were found to be repaired through either non-homologous end joining (NHEJ) or homology-directed repair (HDR) [15]. At the same time, the CRISPR/Cas9 system targeting AAVS1 (Adeno-associated virus integration site 1) was applied to cell lines and induced pluripotent stem cells of another group [16]. Remarkably, the simultaneous introduction of two sgRNAs resulted in the deletion of the fragment between the two sgRNA binding sites, which demonstrated multiplexed genome editing.

Since then, the CRISPR/Cas9 system revolutionized the scientific field. The breakthrough idea that emerged from small discoveries permitted Emmanuelle Charpentier and Jennifer A. Doudna to receive the Nobel Prize in Chemistry 2020 for their development of a method for genome editing. This prestigious award is the cherry on top after years of studies that began from serendipity and resulted in the discovery of an extremely powerful genome editing tool.

## 2. Delivery of CRISPR/Cas9 into Target Cells

**Figure 1:**
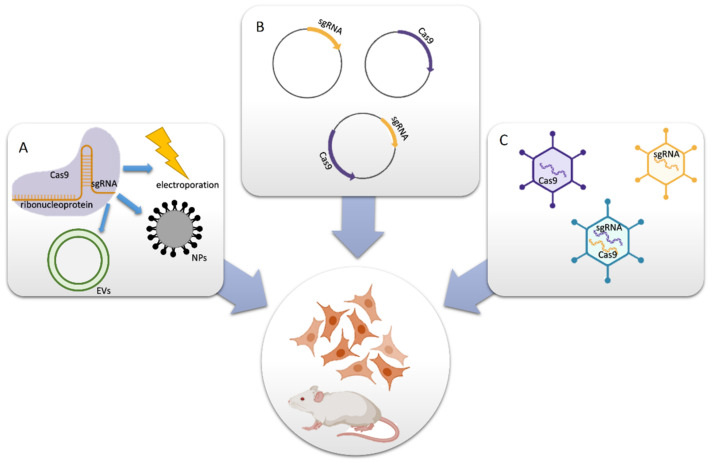
Methods for the delivery of CRISPR/Cas9 components. (**A**) Cas9 protein and sgRNA form a ribonucleoprotein (RNP) complex, which is packaged into extracellular vesicles (EVs), nanoparticles, or electroporated directly into cells or model organisms. (**B**) Plasmids expressing Cas9 and/or sgRNA are transfected into cells. (**C**) Viral vectors encoding Cas9 and/or sgRNA deliver these components in vitro or in vivo. This figure was created using BioRender.com (accessed on April 2021).

The CRISPR/Cas system offers great therapeutic potential. However, it requires finding a golden mean that combines safe delivery and effective gene editing. The delivery method determines genome modification efficiency as well as the frequency of undesired off-target effects. Cas9 and guide RNAs can be delivered in form of DNA, RNA/mRNA, or ribonucleoprotein (Figure 1). The delivery methods are usually divided into physical (electroporation and microinjection), viral (lentiviral, adenoviral, and AAV vectors), and non-viral (plasmids, lipid and polymeric nanoparticles, and extracellular vesicles) ones. Microinjection and electroporation provide a high yield of transfected cells. However, the harmfulness of these techniques restricts their suitability to the modification of zygotes or to ex vivo experiments [17].

An alternative approach uses vectors as delivery agents. Both viral vectors and plasmids can deliver *Cas9* gene and gRNA simultaneously or separately. Despite their effectiveness, each system has its drawbacks. One of the obstacles is the relatively high protein molecular weight (~160 kDa) and gene length (~4.2 kDa) of the SpCas9 endonuclease. AAV vectors possess limited capacity (≤4.7 kb), which is mostly used up by the *SpCas9* and hinders additional modifications. Although AAV vectors are usually maintained in an extra-chromosomal form, high levels of AAV integration were found in CRISPR-induced double-strand breaks (DSBs) in vitro and in vivo. Adenoviruses and lentiviruses can contain both Cas9 and sgRNA in a single vector, therefore, the application is more straightforward. However, the prolonged expression of CRISPR components and integration into the host genome causes more frequent off-target effects. Regrettably, all viral vectors induce immune responses [17,18,19,20,21,22].

A popular approach for in vitro studies is the transfection of cells with plasmids encoding *Cas9* and guide RNA. However, plasmid DNA tends to integrate into the host genome, which increases off target gene editing. Plasmids can also induce immune responses and trigger cyclic GMP-AMP synthase activation in transfected cells [23].

An alternative approach is the usage of ribonucleoproteins (RNPs), which are complexes of Cas9 protein and guide RNA. RNPs cleave targeted sequences promptly after delivery and, contrary to stable transfection, are degraded afterwards. The mutation frequency reaches a plateau one day after delivery and the Cas9 protein is later undetected. Despite its short-term activity, the efficiency of RNPs-mediated indel (insertion or deletion of bases in the genome) generation is comparable to plasmid transfection in cancer cell lines. Interestingly, human primary cells and pluripotent stem cells were less susceptible to plasmid transfection than to electroporation with RNPs. The efficiency of indel formation was two-fold higher in the *CCR5* (C-C chemokine receptor type 5) gene of human fibroblasts and embryonic stem (ES) cells after RNP delivery than compared to plasmid transfection (~20% vs. ~10%, respectively). Moreover, twice as many ES cells were viable after RNP delivery, which indicates that this method is less cytotoxic. Another advantage of RNP usage is the reduction in off-target mutations. The ratio of on-to off-target effects in K562 cells was 2.65-fold to 14-fold higher when RNPs were delivered than compared to plasmids [23].

A comparison of Cas9 delivered as plasmid DNA, mRNA, or RNP was conducted in rat glioma cells, mouse neuronal cells, and mouse and rat single-cell embryos. RNPs were found to be the most effective form for transferring CRISPR components [24]. A similar conclusion was drawn after the plasmid-mediated, lentiviral-mediated, RNA-mediated and RNP-mediated delivery of CRISPR/Cas9 system into human hematopoietic stem and progenitor cells. RNPs provided the best efficiency without inducing cytotoxicity. In contrast, lentiviral transfection was not as effective, but caused the most off-target events [25].

RNP can be delivered via electroporation or using nonviral vectors. Synthetic vehicles such as nanoparticles have recently become more popular due to their non-immunogenicity, lower toxicity, and possible functionalization for targeted delivery. Liposomes have been widely used for nucleic acid delivery in gene therapy. Cationic lipids form lipoplexes with DNA or RNA based on electrostatic interactions. Moreover, anionic proteins can be delivered by cationic lipid reagents. Cas9:sgRNA complexes targeting an EGFP reporter gene were introduced using the cationic lipid formulation RNAiMAX to inhuman bone osteosarcoma epithelial cells. The utilization of the lipid-based reagent was more efficient than plasmid transfection, which caused 60% vs. 38% of indel formation, respectively [26].

The CRISPR-Gold system is dependent on gold nanoparticles for delivering RNP and donor DNA. Its application induced homology directed repair (HDR) in 11.3% of HEK cells. Moreover, it induced HDR in 3–4% of human embryonic stem cells, human induced pluripotent stem cells, primary bone marrow derived dendritic cells, and primary myoblast from mdx mice that are used to study Duchenne muscular dystrophy. CRISPR-Gold was significantly more effective than lipofectamine or nucleofection, while simultaneously causing minimal toxicity. Remarkably, CRISPR-Gold corrected 5.4% of the mutated dystrophin gene in mdx mice (a transgenic mouse model for Duchenne muscular dystrophy) and restored protein expression in muscles, which renders it a very promising therapeutic approach for Duchenne muscular dystrophy treatment [27].

Aside from delivery vectors, CRISPR components can also be immunogenic. The most commonly used form of Cas9 is SpCas9, which originates from *Streptococcus pyogenes*. *S. pyogenes* is a pathogen causing a variety of diseases including pharyngitis, skin infections, and rheumatic fever. Approximately 20% of children are asymptomatic carriers of *S. pyogenes*. Therefore, preexisting adaptive immune responses may be widely present. SpCas9-reactive T cells were found in 96% of donors after stimulation. Anti-SpCas9 and anti-SaCas9 (Cas9 derived from *Staphylococcus aureus*) antibodies were found in 58% and 78% of donors, respectively [28,29].

Moreover, it is worth mentioning that sgRNA can be cytotoxic depending on the synthesis method. Guide RNA can be obtained by chemical synthesis or it can be in vitro transcribed using, e.g., T7 phage RNA polymerases. Chemical synthesis is a more expensive approach, while in vitro transcription is more cost-effective since the yield is much higher. On the other hand, in vitro transcribed sgRNA contains a 5′ triphosphate moiety, which induces immune responses mediated by type I interferons leading to the activation of antiviral effector proteins and cell death in human cells. Chemically synthesized sgRNAs with a 5′-hydroxyl group do not induce innate immune responses. In order to improve the result, in-vitro transcribed sgRNA can be treated with calf intestinal phosphatase [30].

## 3. Extensions of the CRISPR/Cas9 System

**Figure 2:**
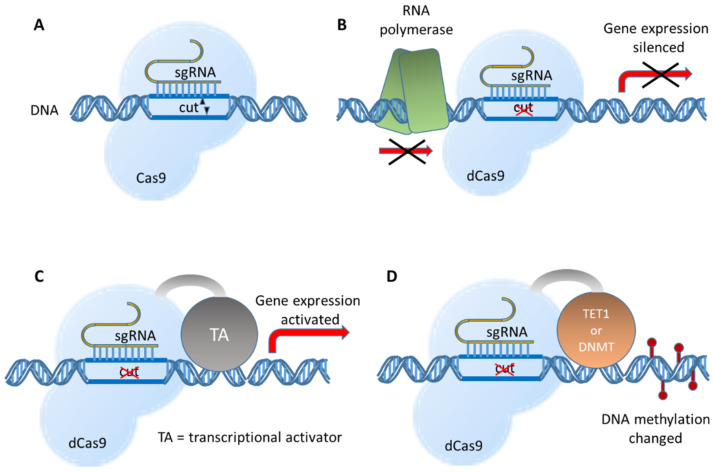
The basic CRISPR/Cas9 system and some examples of further developed fusion enzymes. (**A**) In its basic form, the CRISPR/Cas9 system introduces a double strand break close to the binding site of the sgRNA. (**B**) A mutated Cas9 protein without DNA cutting activity (dead Cas9 and dCas9) is still able to bind to DNA at the specific sgRNA-guided site and blocks the progression of the RNA polymerase, which results in the inhibition of transcription. (**C**) Fused to transcriptional activators, gene expression can be switched on or enhanced at targeted sites. (**D**) The dCas9 fused to histone modifiers or DNA methylation enzymes can be used to introduce site-specific epigenetic changes.

Modifications of Cas9 endonuclease may alter the functioning of the CRISPR/Cas9 system (Figure 2). “Dead” Cas9 (dCas9) for which endonuclease activity is non-functional due to D10A and H840A mutations cannot induce double-strand DNA breaks, but it is still capable of DNA binding. Targeting dCas9 to transcription start sites physically blocks RNA polymerase movement and hinders transcription, thus silencing gene expression through CRISPR interference (CRISPRi) [31].

In order to improve the effectiveness of CRISPRi, *dCas9* is often fused with effector domains. Fusing *dCas9* with a *KRAB* (Krüppel associated box) repressor domain and methyl-CpG binding protein 2 (*MeCP2*) increased specific gene silencing [32]. Employing *dCas9-KRAB-MeCP2* suppressed luciferase reporter gene expression in HEK293 cells more efficiently (83.3%) than employing *SYN-dCas9* (17.8%) or *SYN-KRAB-dCas9*/*EGFP* (68.2%) that contained the neuron-selective human synapsin 1 promoter (*SYN*). Fusion constructs incorporating SYN achieved gene silencing in neurons of multiple targets, including lysine methyltransferase *Kmt2b*, extracellular matrix protein *Reln*, signaling neuropeptide *Npy*, and brain-derived neurotrophic factor *Bdnf* in primary rat hippocampal cultures [33].

On the other hand, transcriptional activators fused to dCas9 can upregulate gene expression (CRISPR activation and CRISPRa). Increased expression of several genes, including *VEGFA* (Vascular Endothelial Growth Factor A), *NTF3* (Neurotrophin 3), and *IL1RN* (Interleukin 1 Receptor Antagonist), was induced in HEK293 cells transfected with plasmids encoding sgRNA and dCas9 fused with the activation domain VP64 (a concatemer of the herpes simplex viral protein VP16) [34,35]. Optimized for the expression in neurons, dCas9 was fused to three enhancer proteins, VP64, the p65 subunit of the transcription factor NF-κB, and Rta (a herpesvirus transactivator) and put under the control of the neuron-specific promoter SYN. Lentiviral vectors expressing *dCas9-VPR* (*VP64-p65-Rta*) and sgRNA targeting an array of genes important for neuronal development and memory functions were stereotaxically infused into the rat’s hippocampus, nucleus accumbens, or prefrontal cortex. Increased gene expression in vivo in male rats was achieved as exemplarily shown by Fosb (FosB Proto-Oncogene) [36].

Another application of CRISPR is the introduction of epigenetic changes by combining epigenetic effectors with targeted DNA binding domains. Epigenome engineering strategies include chromatin editing, namely histone acetylation and methylation and DNA methylation [37]. Remarkably, the epigenetic changes are maintained through cell divisions [38]. Dead Cas9 proteins fused to enzymes mediating DNA methylation of repressive histone modifications established the CRISPRoff system able to silence most genes in a heritable manner. The epigenetic modifications were even kept when human iPSCs (induced pluripotent stem cells) were differentiated into neurons [38]. In addition, the CRISPRoff tools can be used to study general rules for heritable gene silencing.

In another study [39], human colon cancer cell line HCT116 cells were co-transfected with plasmids expressing sgRNAs targeting the *HER2* (human epidermal growth factor receptor 2) promoter, DNA (cytosine-5)-methyltransferase 3-like (*D3L*), dCas9 fused with DNA cytosine-5-methyltransferase 3A (*dCas9-D3A*), and either dCas9-H3K27 (histone 3 lysine 27) tri-methyltransferase (*dCas9-Ezh2*) or dCas9-H3K9 tri-methyltransferase (*dCas9-KRAB*). When *dCas9-Ezh2* combined with *dCas9-D3A* and *D3L*, it induced the two-fold repression of HER2 expression in 31% of HCT116 cells lasting 50 days. On the contrary, *KRAB*-mediated repression was short-term and 50 days after the transfection HER2 silencing was observed in only 2% of cells. *dCas9-Ezh2* + *dCas9-d3A* + *D3L* and *dCas9-KRAB-dCas9-d3A* + *D3L* increased methylation by 54% or 39%, respectively. Moreover, hypermethylation was spread over 12 CpG islands in the *HER2* promoter region after *dCas9-Ezh2* treatment, while *dCas9-KRAB* induced hypermethylation in only one CpG cluster downstream the targeted region. Additionally, a 4.7-fold increase in H3K27 trimethylation and a 5.2-fold decrease in H3K27 acetylation was observed 24 days after *dCas9-Ezh2* transfection, while *dCas9-KRAB* initially induced a 102-fold increase in H3K9 trimethylation, but the effect was temporary. The *dCas9-KRAB* + *dCas9-d3A* + *D3L* combination was able to repress *HER2* in LNCaP (Lymph Node Carcinoma of the Prostate) cells up to two weeks after transfection [39]. Interestingly, the long-term effectiveness of these epigenetic effector domains was locus and cell specific.

Finally, the CRISPR/Cas9 functionality was extended to allow single base substitutions. Such base editors is composed of a Cas9 nickase that is able to introduce single strand cuts and an enzyme with nucleotide deaminase activity [40,41,42]. SgRNA-directed binding of the Cas9 protein opens up the double helix and allows access for cytidine deaminase base editors (CBEs) or adenosine deaminase base editors (ABEs) to deaminate C-to-U or A-to-I, respectively. The Cas9 nickase creates a nick in the unedited strand that stimulates DNA repair. As the deaminated base is used as template for DNA polymerization, the new U base pairs with A and the new I base pairs with C, thus creating C:G to T:A and A:T to G:C changes. Further development resulted in a base editor suitable for concurrent A-to-G and C-to-T substitutions [43,44]. In a preclinical study, application of an ABE corrected a point mutation in the *TERT* (telomerase reverse transcriptase) promoter that is found in many types of cancer. As a consequence, *TERT* transcription and TERT protein levels were reduced resulting in human glioblastoma cell senescence. Packed into adeno-associated viral particles, this sgRNA-guided ABE was able to inhibit the growth of gliomas in mice [45]. Along with the basic CRISPR/Cas9 mechanism to knockout specific genes, BE-mediated disruption (CBEs and ABEs) of splice donor and acceptor sites can yield similar results with excellent efficiency [46].

The most advanced technique was brought by the so-called ‘prime editors’ that allow all 12 possible base exchanges in addition to small insertions or deletions [47]. Prime editing uses a reverse transcriptase fused to the Cas9 nickase and special guide RNAs (pegRNAs and prime editing guide RNAs) that contain the primer binding site together with a RT template sequence. Even insertions (up to 40 bp) and deletions (up to 80 bp) can be performed without double strand breaks.

## 4. Major CRISPR/Cas9 Applications

The CRISPR/Cas9 system is a versatile tool that can be applied in many fields, from basic science to biotechnology and medicine. Since its discovery, Cas9 endonuclease was administered to in vitro cell cultures as well as organisms including rodents, zebrafish, fruit flies, plants, bacteria, and yeast [48,49].

Knocking out genes enables the determination of their function and physiological role. Genome editing used for disease modeling permits the understanding of the etiology of diseases and explores the mechanisms of pathogenesis, which may lead to discovering new therapeutic targets. For example, the CRISPR/Cas9 system was used to introduce mutations in the tumor suppressor genes *APC* (Adenomatous polyposis coli), *SMAD4* (SMAD Family Member 4), and *TP53* (Tumor protein P53) and the oncogenes *KRAS* (KRAS Proto-Oncogene) and *PIK3CA* (Phosphatidylinositol-4,5-Bisphosphate 3-Kinase Catalytic Subunit Alpha) in organoids derived from normal or adenoma human intestinal epithelium. Organoids with multiple modifications formed tumors in mice and permitted the determination of the contribution of single pathway mutations in human colorectal carcinogenesis [50].

Genome wide loss-of-function screening using sgRNA libraries can be performed to identify multiple genes involved in biological processes, including the development of drug-resistance. For instance, the human KBM7 CML (Chronic myeloid leukemia) cell line expressing Cas9 was transduced with a lentivirus library containing sgRNA targeting 7114 genes and 100 non-targeting controls. The resulting mutant pools were screened for resistance to treatment with the nucleotide analogue 6-thioguanine (6-TG). Massive parallel sequencing supported by sgRNA barcodes revealed the enrichment of sgRNAs targeting *MSH2* (MutS Homolog 2), *MSH6* (MutS Homolog 6), *MLH1* (MutL Homolog 1), and *PSM2* (PMS1 Homolog 2) genes that are involved in DNA mismatch repair. Loss-of-function screening was also performed in HL60 cells treated with etoposide, a topoisomerase inhibitor. SgRNA targeting *TOP2A* (DNA topoisomerase II alpha) and *CDK6* (Cyclin dependent kinase 6) were strongly enriched and this indicates that the loss of these genes provided resistance to etoposide [51].

A sgRNA library targeting 18,080 genes was cloned into lentiviral vectors to transfect human melanoma cells (A375) and human stem cells (HUSES62). In the negative selection screening, genes essential for cell viability were identified. The most deleterious sgRNA targets were essential genes involved in RNA processing and binding, ribosome structural constituents, etc. The same library was then used for a positive screening approach. Transfected melanoma cells were treated with vemurafenib, which is a BRAF (B-Raf proto-oncogene) protein kinase inhibitor. Enrichment of sgRNAs permitted the identification of previously known (*NF1* (Neurofibromin 1) and *MED12*—Mediator complex subunit 12) and new genes (*NF2* (Neurofibromin 2), *CUL3* (Cullin 3), *TADA1* (Transcriptional adaptor 1), and *TADA2B*—Transcriptional adaptor 2B) associated with vemurafenib-resistance [52]. An optimized minimal genome-wide human CRISPR-Cas9 library (MinLibCas9), which targets 18,761 genes but reduced the number of sgRNAs by 42%, was recently created based on previously published data [53].

CRISPR interference was also used to identify long non-coding RNAs (lncRNAs) that are regulated by the oncoprotein *MYC* (MYC proto-oncogene). The deregulation of lncRNAs has been associated with cancer progression. The human lymphoid cell line P493–6 and the Burkitt’s lymphoma cell line RAMOS were transduced with lentiviral vectors to stably express dCas9 fused with the SIN3 interacting domain of *MXD1* (MAX Dimerization Protein 1). A sgRNA library targeting transcription start sites of 508 non-coding and 100 coding genes regulated by MYC as well as 14 essential genes was subsequently delivered. Screening revealed that individual depletion of several lncRNAs such as DNM2, RAD51-AS1, tTN-AS1, SNHG17, and ZNF433-AS1 inhibited cell growth, thus confirming their role in the proliferation of cancer cells [54].

One of the most exciting CRISPR applications is potential human gene therapy. Fusion oncogenes caused by translocation of two genomic regions are common and exclusive to cancer cells, which makes them a promising therapeutic target. For instance, the *EWSR1-FLI1* fusion that occurs in Ewing sarcoma was targeted by an all-in-one plasmid containing Cas9 and two guide RNAs. The EWSR1-FLI1 fusion protein acts as a dominant transcription factor rendering the cancer cells addicted to the expression of this protein. The elegance of the system lies in the fact that CRISPR/Cas9-induced deletion of the oncogene occurs only in cells containing the fusion. Targeting introns in the two fused genes generated large deletions mediated by NHEJ. This approach was first tested in human Ewing sarcoma cell lines in which inhibition of cell proliferation and clonogenicity was observed. Furthermore, tumor growth was also inhibited in patient-derived xenografted mice [55]. For validation of the approach, another fusion gene, BCR-Abl, was chosen and it drives chronic myeloid leukemia (CML). Transfection of the BCR-Abl expressing CML cell line K562 with a plasmid coding for appropriate sgRNAs and Cas9 abrogated expression of the fusion protein. In mouse xenograft experiments, the application of the deletion strategy inhibited tumor growth by up to 88% [55].

CRISPR/Cas9 is a powerful tool that could revolutionize the therapy of genetic diseases (please refer to [56,57] for recent reviews). The most important genetic disorders apart from cancer that are targeted by CRISPR/Cas9 based approaches include Duchenne’s muscular dystrophy, Cystic fibrosis [58], Leber congenital amaurosis, β-Thalassemia [59], Sickle-cell disease, Huntington’s disease, and HIV (human immunodeficiency virus).

Base editors are capable of correcting single-nucleotide polymorphisms by converting single DNA bases. Nickase-Cas9 fused to a cytidine deaminase and base excision repair proteins were able to create single base C:G to G:C transversions. This approach was applied to target genes associated with dyslipidemia (ADRB2), hearing loss (GJB2), and hypertrophic cardiomyopathy (MYBPC3) in HEK293 cells [60].

Furthermore, the CRISPR/Cas9 system was applied to treat Wolfram syndrome (WS), an autosomal recessive disorder which causes childhood-onset diabetes, optic atrophy, neurodegeneration, deafness, etc. WS is caused by mutations in the *WFS1* (Wolframin ER transmembrane glycoprotein) gene which leads to chronic endoplasmic reticulum (ER) stress and the impeded folding of proinsulin. Induced pluripotent stem cells (iPSCs) collected from patients’ fibroblasts were edited by CRISPR-mediated homology-directed repair to correct point mutations. Human iPSCs were later differentiated into stem cell-derived pancreatic β cells (SC-β cells). Both unedited and corrected SC-β cells secreted insulin. However, only edited cells dynamically responded to changing glucose concentrations. Gene-edited cells were transplanted to mice with streptozotocin-induced diabetes. Mice transplanted with corrected cells maintained normal levels of blood glucose and higher insulin concentration for 10 weeks, which confirmed that gene editing of differentiated stem cells successfully reversed diabetes. Moreover, the correction of the *WFS1* mutation decreased the expression of stress and apoptotic markers in ER and mitochondria [61].

Hemophilia A is a monogenic disorder caused by a mutation in the factor VIII (*F8*) gene, which leads to a deficiency of the blood clotting factor VIII (FVIII) [62]. Therapies are based on supplementation of FVIII. The CRISPR/Cas9 system was applied to insert a modified human B domain deleted-F8 (BDD-F8) in a site-specific manner at the albumin (*Alb*) locus in murine liver cells. Due to the fact that full F8 DNA sequence exceeds the packaging capacity of AAVs, the B-domain, which was found to be uncritical for pro-coagulation activity of FVIII, was removed. The AAV vectors expressing SaCas9 and gRNA targeting Alb intron 13 and a donor vector AAV8-BDD-F8 were administered intravenously to healthy (C56BL/6) and FVIII knockout (F8KO) mice. BDD-F8 was inserted at the Alb locus by NHEJ-mediated knock-in with approximately 0.2–0.3% efficiency. Human FVIII expression was successfully induced in mouse hepatocytes and restored ~13% procoagulant activity. The therapeutic effects lasted for at least 7 months after the injection [62].

Another approach for CRISPR/Cas9 based gene therapy corrected a nonsense mutation in Rpe65 that is associated with Leber congenital amaurosis (LCA) [61]. LCA is a hereditary retinal degenerative disease and it causes blindness in childhood. A dual AAV system consisting of AAV expressing *SpCas9* and *AAV-TS4rd12* sgRNA-Rpe65-donor was injected subretinally to rd12 mice that carried a premature stop codon in Rpe65. The indel formation frequency in retina and retinal pigment epithelium was approximately 20%. The precise correction of the T-to-C mutation, which generates the premature stop codon, was obtained via HDR with ~1% frequency. The Rpe65 expression was increased 6 weeks after the injection and lasted for 7 months. Consequently, the retinal function was improved and the retinal degeneration was stopped [63].

Several clinical trials using CRISPR/Cas9 are currently under way, including CRISPR/Cas9 mediated treatment for transfusion-dependent β-thalassemia (TDT) and sickle-cell disease (SCD), which are among the most common monogenic diseases and affect oxygen transport in the blood [64]. In order to induce expression of fetal hemoglobin, human CD34+ hematopoietic stem and progenitor cells (HSPCs) from healthy donors were electroporated with CRISPR/Cas9 targeting an erythroid-specific enhancer region of *BCL11A*, a transcription factor repressing fetal hemoglobin expression. The edited CD34+ HSPCs displayed reactivation of the production of fetal hemoglobin. One patient with TDT and another one with SCD were infused with the modified CD34+ cells after undergoing myeloablation and were monitored for over a year. The gene editing frequency was in the range of 70% to 80%. Fetal hemoglobin levels increased pancellularly and lasted for at least one year. Both patients suffered from adverse events including pneumonia, sepsis in the presence of neutropenia, cholelithiasis, and abdominal pain. These initial results confirmed that CRISPR/Cas9 editing of *BCL11A* in engrafted HSPCs successfully increased fetal hemoglobin expression and reduced vaso-occlusive episodes and the need for transfusion (clinical trial NCT03655678, [64]).

One of the therapeutic strategies for combating HIV infection is targeting *CCR5*, which acts as a co-receptor to HIV-1 entry. This concept appeared because of the “Berlin Patient”, who was the first person that was cured of HIV as a result of hematopoietic cell transplant from the donor who carried a naturally occurring Delta32 (Δ32) mutation in the *CCR5* gene [65]. Artificial disruption of *CCR5* became a promising method to generate resistance to HIV-1 infection (see below) and the application of the CRISPR/Cas9 technology for that purpose is currently tested in a clinical trial (Hu, C. Safety of transplantation of CRISPR CCR5 modified CD34+ cells in HIV-infected subjects with hematological malignances. https://clinicaltrials.gov/ct2/show/NCT03164135, accessed on 20 May 2021).

## 5. Extracellular Vesicles

Extracellular vesicles (EVs) are heterogeneous populations of membrane vesicles released by all kinds of cells. Three types of EVs can be distinguished based on their size and biogenesis [66]. Exosomes, as the smallest vesicles with 40–150 nm diameters, are of endocytic origin. Microvesicles (ectosomes), with a diameter of 100–1000 nm, emerge from the plasma membrane by outward budding. The apoptotic bodies, the third class of EVs, are produced during the fragmentation of apoptotic cells and come with diameters ranging from 50 up to 5000 nm. Despite the different modes of production, all these vesicles own a lipid bilayer membrane with the same topological orientation as the plasma membrane [67].

Exosomes are constantly released by most cells and can be found in various biological fluids including blood plasma, urine, breast milk, and saliva. They function as carriers of information, e.g., in form of messenger RNA (mRNA) and miRNA that can be transferred from the producer to the target cells [68]. They are formed by inward budding of the membrane of the multi-vesicular body (MVB), which is an endosomal compartment. When MVBs fuse with the plasma membrane, the exosomes are secreted and they interact with the target cells in the close neighborhood or enter the circulating fluids of the body to reach more distant targets. Exosomes may gain entry into a target cell via various forms of endocytosis, or through fusion with the plasma membrane [69]. Internalized exosomes are either degraded or they deliver their content into the cytosol. During vesicle formation, various proteins, lipids, and nucleic acids are inserted into exosomes in a controlled manner [70,71]. Consequently, their RNA and protein content differs from the molecular composition of the producer cell. However, there is also a sufficient overlap of the molecular patterns of exosomes and their producer cells to allow for the identification of the producer cell type by analyzing the exosomal composition. This fact is utilized for diagnosis of a multitude of diseases (“liquid biopsies”), a procedure with enormous potential and is currently rapidly increasing clinical implementation [72,73,74].

Exosomes constitute a promising tool for the delivery of therapeutic molecules. They offer major advantages as drug delivery vehicles as they exhibit the natural ability to carry intercellular nucleic acids and therapeutic molecules across biological membranes. Various methods of drug loading into exosomes have been described, including electroporation (the most common), membrane permeabilization with detergents, freeze–thaw cycles, sonication, or extrusion [75].

In addition to exosomes, microvesicles (MVs) have also been characterized to alter recipient cells by transporting proteins, lipids, and various kinds of RNA, such as miRNA, mRNA, and long non-coding RNA (lncRNA) [76]. They might even promote invasion and metastasis of breast cancer cells [77]. MV release is increased when cancer cells become invasive and leave firm matrices [78]. MV secretion is regulated by the small GTPases ARF6 and RhoA through phosphorylation of the myosin light chain [79,80].

While exosomes and microvesicles can be clearly distinguished theoretically based on their biogenesis, they may have similar size and physical properties and so their experimental separation is very challenging [81]. Most of the time, EVs isolated from various sources will constitute a mixture of both kinds of vesicles. Therefore, it was suggested to replace the terms “exosomes” and “microvesicles” with “small” and “large extracellular vesicles” (sEVs and lEVs), respectively, depending on their diameter above or below 200 nm [82].

## 6. Comparison between EVs and Synthetic Nanoparticle for the Delivery of Therapeutics

The development of synthetic carriers that meet all requirements for efficient delivery of nucleic acids and other therapeutic molecules into particular tissues in vivo remains very challenging. These requirements include: efficient production of the vehicle and cargo loading, protection against degradation, lack of immunogenicity, precise targeting, sufficient cellular uptake, no/low cytotoxicity, release of the cargo into the correct intracellular compartment, and mediating the desired effects [83]. Synthetic transfection carriers exhibit a huge advantage, which is that their properties can be specifically tailored to meet these requirements. However, most of these properties are not independent from each other and improving one feature can introduce unwanted deficiencies. For example, increasing the biodegradability to reduce toxicity could lower the serum half-life; the improved mechanisms for release into the right cellular compartment could increase cytotoxicity; in case of RNA transport, stronger binding of the RNA molecule to the carrier can provide better protection from degradation but could simultaneously decrease the release rate inside the cell.

The usage of sEVs as a delivery system avoids many of the above problems, as EVs have been optimized by evolution and displays a harmonic balance of the requirements for being a good transfer vehicle. However, major problems with EVs consist in large scale production, adequate purification, and efficient loading with cargo. The successful clinical translation of sEV-based nanomedicines still has to overcome serious obstacles [84]. In addition to the difficulties associated with producing EVs in such large quantities required for therapeutic applications, the isolation and purification needs to be standardized to meet the Good Manufacture Practice (GMP) guidelines. As EVs are extremely complex structures and present with large inter-vesicular heterogeneity, adjusted rules are required to guarantee their safe application. Furthermore, loading efficiency of isolated EVs varies widely and has to be substantially improved, together with targeting capabilities.

A quantitative comparison of the efficiency of RNA delivery between EVs and synthetic nanoparticles was recently published [85]. Capitalizing on a sensitive reporter system based on CRISPR/Cas9, the actual activity of the transferred RNA inside of the target cell was measured. This system called “CRISPR Operated Stoplight System for Functional Intercellular RNA Exchange” (CROSS-FIRE, please find a more detailed description in Section 8) was described by the same research group in a previous publication [86]. When the CROSS-FIRE system was used to compare EVs and synthetic nanoparticles in terms of the efficacy of RNA delivery, EVs outcompeted synthetic nanoparticles by several orders of magnitude [85]. However, rapid technological progress may soon close this gap [87]. Lipid nanoparticles for the delivery of siRNA to the liver were already approved by the American FDA (Food and Drug Administration) [88]. To provide an overview of the huge field of synthetic nanocarriers with the potential to be used for CRISPR/Cas9 component delivery here would go beyond the scope of this article. To give just one example of a recent publication, lipid nanoparticles were demonstrated to efficiently co-deliver Cas9 mRNA and sgRNAs to the liver [89]. By targeting the gene encoding *Angptl3* (Angiopoietin-like 3) to treat a human lipoprotein metabolism disorder, the authors not only showed that the gene knockdown was liver-specific and reduced the serum levels of ANGPTL3 protein but also reduced triglycerides and low-density lipoprotein cholesterol. Remarkably, a single dose administration resulted in a therapeutic effect for more than 100 days.

## 7. General Routes for Loading EVs with CRISPR/Cas9 Components

**Figure 3:**
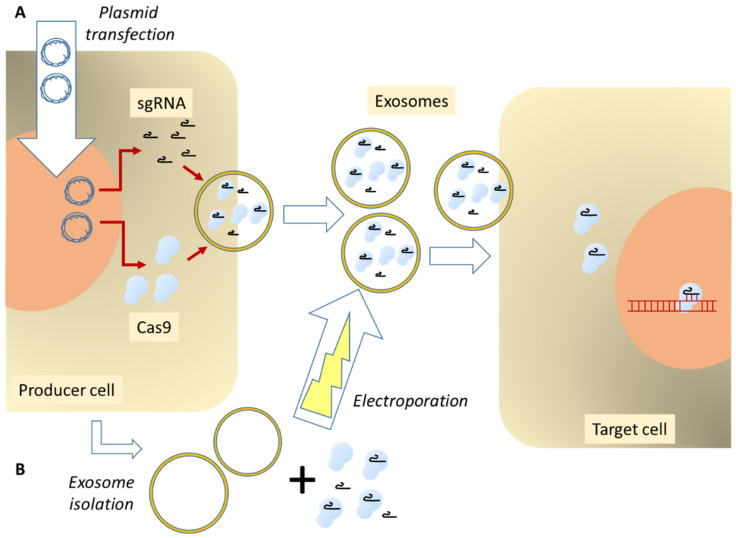
General routes for loading CRISPR/Cas components into EVs. (**A**) By capitalizing on the cellular packaging routines, producer cells can be transfected with plasmids encoding the Cas9 protein and the sgRNA. (**B**) After the isolation and purification of EVs, they are loaded in vitro with Cas9 protein and sgRNAs, e.g., by electroporation.

The loading of CRISPR components into EVs can be accomplished either by using purified sEVs or exploiting the cellular packaging mechanisms (Figure 3).

A modern functional CRISPR/Cas9 unit consists of one RNA molecule associated with the Cas9 protein. Both parts can be encoded by DNA and introduced into cells in the form of plasmids or viral vectors. Alternatively, both components can be introduced as RNA molecules or as preformed protein/RNA complexes. In the following chapters we will present a few publications that used one or the other possibilities, focusing on the delivery of Cas9 protein in combination with guide RNAs as the most interesting recent development. Table 1 presents a short summary of advantages and disadvantages of producer cell-based EV engineering versus the manipulation of isolated EVs.

## 8. Producer Cell Based Exosome Engineering

Bioengineered cells can be a source of exosomes containing sgRNA and Cas9 protein. This approach was tested in an antiviral application of CRISPR/Cas9 targeting human hepatitis B virus (HBV) and human papilloma virus (HPV) [90]. Various cells lines were transfected with anti-viral guide RNAs targeting HPV or HBV and Cas9 plasmids. Exosomes isolated from the cell culture media contained HPV or HBV specific guide RNA and Cas9 protein which was confirmed by RT-PCR, Western blotting, and sequencing. Plasmid DNA was undetectable by PCR. Moreover, Cas9 and gHPV1 could be carried by exosomes independently, which was confirmed by transfecting producer cells with plasmids coding for Cas9 or gRNA only. Co-culture of the donor cells with HBV transfected HuH7 cells resulted in the cleavage of virus DNA and a suppression rate of ~30% of HBV replication, which demonstrates that bioengineering cell lines can be used for producing exosomes as carriers of gRNA/Cas9 for gene editing activity. Confocal microscopy showed the entry of the secreted exosomes into HuH7 cells. To prove the involvement of EVs, GW4869, an inhibitor of exosome secretion, was added six hours post transfection. HBV replication suppression mediated by CRISPR/Cas9 was significantly diminished (to less than 10%), which demonstrates that exosomes were the major players in the intercellular delivering the CRISPR/Cas9 system [90].

The CROSS-FIRE system provides a modern, versatile, and powerful tool for employing EVs for multiple purposes [86]. The method is based on a fluorescent “Stoplight” reporter system, which contains constitutively expressed *mCherry* and a linker between mCherry and its stop codon. Targeted cleavage by Cas9 in the linker region causes a frame-shift mutation, which bypasses the stop codon and enables eGFP expression. Reporter cells were transduced with lentivirus to generate Stoplight + spCas9+ reporter cell line stably expressing spCas9. The transfection of plasmids expressing sgRNA targeting the linker region of the Stoplight construct resulted in the activation of eGFP expression. To demonstrate the transfer of sgRNA by EVs, a donor cell line stably expressing sgRNA was generated and co-cultured with reporter spCas9+ cells for five days. SgRNA, transferred from donor cells, coupled with Cas9 expressed by reporter cells activated the eGFP expression and this was confirmed by flow cytometry and fluorescence microscopy. Moreover, the percentage of eGFP+ cells increased in a dose-dependent manner when using different donor:reporter cell ratios. These experiments were carried out in several reporter (HEK293T, HeLa, HMEC-1, MCF-7, and MDA-MB-231) and donor cell lines (HEK293T, MDA-MB-231, and TERT-MSC). Transwell co-culture assays confirmed that cell contact was not required for the intercellular transfer of sgRNA and excluded delivery through cell–cell fusion. Therefore, EVs secreted by donor cells were acknowledged as vehicles delivering RNA. This was additionally confirmed by inhibiting EV production with GW4869, which decreased the reporter activation. RT-PCR showed that EVs isolated from sgRNA+ MDA-MB-231 donor cells contained sgRNA that was also protected from RNase degradation. Uptake of EVs labelled with the fluorescent PKH67 lipid-dye by reporter Stoplight+ HEK293T cells was imagined on a confocal microscope.

CROSS-FIRE was employed to clearly identify specific genes and pathways involved in EV-mediated RNA transfer [86]. SiRNA knockdown of genes involved in exosome biogenesis (*Alix*, ALG-2 interacting protein X) and release (*Rab27A*, member RAS oncogene family) in donor cells decreased reporter activation in recipient cells, while the knockdown of *ARRDC1* (Arrestin domain containing 1), involved in release of microvesicles, did not affect it. Moreover, several genes, namely Rho GTPases *Rac1* (Rac Family Small GTPase 1) and *RhoA* (Ras Homolog Family Member A) and the Rho GTPase effector *PAK1* (p21 (RAC1) activated kinase 1) and *Cav1* (Caveolin 1), were identified as genes crucial for EV-mediated RNA transfer determined by knocking down genes responsible for endocytosis and intracellular membrane trafficking in HEK293T. Interestingly, Rho GTPases CDC42 (Cell division cycle 42) and ANKFY1 (Ankyrin repeat and FYVE domain containing 1) involved in intracellular vesicle transport and Flot-1 (Flotillin 1) engaged in endocytosis did not affect RNA transfer. Experiments in different cell lines indicated that the pathways of EV-mediated RNA transfer are cell type dependent.

The CROSS-FIRE system can further be used to study EV uptake. Reporter cells were transfected with *Cav1*-targeting siRNA and then treated with sgRNA+ EVs isolated from donor cells. Cav1 siRNA prevented eGFP expression by inhibition of EV uptake. Decreased eGFP activation was also observed after knocking down several genes involved in endocytosis (*ABL*—Abl tyrosine kinase and DIAPH1—Diaphanous related formin 1), extracellular matrix adhesion (*ITGB1*—Integrin subunit beta 1), intracellular membrane trafficking (*Rab4*, *Rab5*, *Rab7*, and *Rab11*), or interaction with Rho GTPases (RhoA effector *ROCK1* (Rho associated coiled-coil containing protein kinase 1) and Rac1 interactors *Tiam1* (TIAM Rac1 associated GEF 1) and VAV2 (vav guanine nucleotide exchange factor 2). Interestingly, CROSS-FIRE permitted the exclusion of genes to be associated with RNA transfer, including *ITGB1*, *Rab5*, *Rab7*, and *ROCK1* [86].

Ye et al. [93] first confirmed that in exosomes sgRNA and Cas9 protein existed as a sgRNA:Cas9 ribonucleoprotein complex. HEK293T cells were transfected with a plasmid encoding sgRNA and *Cas9* from *Streptococcus pyogenes*. Exosomes derived from transfected and control cells had similar diameters (143 ± 2.2 nm and 149 ± 1.2 nm, respectively) measured by nanoparticle tracking analysis. qRT-PCR and DNA agarose gel electrophoresis confirmed that sgRNA and Cas9 protein were packaged into CRISPR/Cas9 exosomes, while Cas9 mRNA levels were undetectable. Plasmids expressing sgRNAs and FLAG-tagged Cas9 proteins were used to determine whether CRISPR/Cas9 components formed a ribonucleoprotein complex in exosomes. FLAG-tagged Cas9 proteins were immunoprecipitated from exosomes derived from transfected cells. Guide RNA was detected by qRT-PCR in the immunoprecipitates of FLAG-tagged Cas9 exosomes pulled down with an anti-FLAG antibody, which confirmed the formation ribonucleoprotein complexes.

Then, the lung adenocarcinoma cell line A549 was treated with normal exosomes and exosomes containing sgRNA and Cas9 protein. Surprisingly, despite the successful loading of RNP complexes in exosomes, gene expression was not efficiently altered by the CRISPR/Cas9 system. Therefore, a modified system was designed to consist of a plasmid expressing exosomal membrane protein CD63 fused with the GFP protein and a plasmid carrying a GFP nanobody (a single-domain antibody) fused with Cas9. GFP nanobodies fused with Cas9 were binding to GFP-CD63 with high affinity and facilitated efficient loading of Cas9 into exosomes. In addition to sgRNA and Cas9 protein levels, the Cas9 mRNA levels in modified exosomes were also significantly increased.

The functionality of modified CRISPR/Cas9 exosomes was studied in a reporter stop-DsRed A549 cell line in which the DsRed fluorescent protein was not expressed due to a stop sequence. SgRNA targeting the stop sequence aimed to restore the red fluorescence. After incubating A549 stop-DsRed cells with CRISPR/Cas9 exosomes, they exhibited weak red fluorescent signals, while the cells treated with modified CRISPR/Cas9 exosomes provided clearly detectable scattered red fluorescence signals. Accordingly, the sgRNA concentration was higher in modified exosomes. The precise removal of nucleotides in the stop sequence was verified by Sanger sequencing [93].

An all-in-one EV delivery system called NanoMEDIC (nanomembrane-derived extracellular vesicles for the delivery of macromolecular cargo) was used for gene editing in vitro and in vivo [94]. The EVs are shed from the plasma membrane of the producer cells that express HIV gag protein potentially giving rise to lentivirus-like particles [103]. The system consists of two Gag-mediated homing mechanisms to package Cas9 protein and sgRNA separately and synergistically. An HIV-derived packaging signal was used to direct sgRNA flanked by hammerhead and hepatitis delta virus self-cleaving ribozymes into nanoparticles through the interaction with Gag. Furthermore, chemically induced dimerization facilitated the recruitment of Cas9 protein into EVs in the producer cells. The dimerization of FKBP12 (FK506-binding protein) and FRB (a small domain from mTOR = mammalian target of rapamycin) was induced by rapamycin. An FRB variant that specifically binds to AP21967, a rapamycin analog, was fused to SpCas9. Three membrane-anchoring proteins VSV-G-FKBP12, LM-FKBP12-Gag containing the myristoylation motif from human Lyn kinase (LM), and LM-FKBP-EGFP were studied.

HEK293T producer cells were transfected with one of the membrane-anchoring FKBP12 variants and FRB-Cas9 by lipofection. After the addition of AP21967, EVs were isolated and added to recipient HEK293T cells expressing a specific sgRNA targeting DMD1 (the SA site of exon 45 in the human dystrophin gene that is mutated in DMD, Duchenne muscular dystrophy). Western blot analysis confirmed the incorporation of SpCas9 protein into EVs, while indel formation in the targeted sequence was detected by the T7E1 assay which relies on the ability of T7 endonuclease I to selectively recognize and cleave indels. The best system of SpCas9 delivery was FKBP12-Gag in the presence of AP21967 leading to the most efficient gene editing in cells expressing sgRNA-DMD1.

When these HEK293T cells were transfected with plasmids to express a EGxxFP reporter, which contains a 100 bp sequence targeting sgRNA-DMD1 in the GFP coding region, DNA cleavage in the targeted sequence restored EGFP expression. In comparison to the commercially available gesicles (see below), NanoMEDIC provided more efficient restoration of EFGP in HEK293T EGxxFP cells.

NanoMEDIC efficiently generated indels in the *DMD1* gene in human undifferentiated Hu5 myoblasts and murine differentiated C2C12 myotubes. Moreover, the functionality of NanoMEDIC was demonstrated in induced pluripotent stem cells (iPSCs). Indels were generated in human iPSCs in a dose-dependent manner in up to 40% of cells. Up to 48% of indels were induced in Jurkat T-lymphocyte cells via nanoMEDIC by targeting the *CCR5* gene (HIV co-receptor). EGFP expression was knocked out in U937 monocyte cells stably expressing EGFP. NanoMEDIC targeting the *SAMHD1* (SAM and HD domain containing deoxynucleoside triphosphate triphosphohydrolase 1) gene, which is accountable for congenital encephalopathy, edited the iPSC-derived cortical neurons with up to 36% efficiency. Interestingly, nanoMEDIC containing RNP targeting six different genomic loci outperformed plasmid DNA transfection.

Targeting the dystrophin gene is one of the main potential applications of NanoMEDIC. CRISPR mediated cleavage of the exon 45 SA site can induce exon 45 skipping and restore dystrophin protein expression in iPSC-derived skeletal muscle cells. Loading nanoMEDIC with sgRNA targeting the SA site (DMD1) or SD site (DMD23) resulted in exon skipping with over 50% frequency. After differentiation of DMD iPSCs lacking exon 44 into skeletal muscle cells, exon skipping efficiency was examined. NanoMEDIC targeting SA induced 36% exon skipping and nanoparticles targeting SD site did not affect it. Surprisingly, multiplexed NanoMEDIC targeting SA and SD lead to synergistic effect, yielding up to 92% exon skipping.

NanoMEDIC did not affect cell viability, but it reduced off-target activity of plasmid DNA mediated CRISPR/Cas9 knockout, which is a major drawback of plasmid transfections. While on target cleavage activity targeting the VEGFA gene in HEK293T cells was comparable—32.5% for NanoMEDIC vs. 31.5% for plasmid DNA—off target activity was almost excluded after NanoMEDIC treatment. The on/off target ratio was over 70-fold for NanoMEDIC vs. 1.8-fold for plasmid DNA. Similar results, 27-fold vs. 4.1-fold ratio was observed for NanoMEDIC and plasmid DNA targeting the EMX1 gene. These results highlight the advantage of the NanoMEDIC system for potential clinical use.

For in vivo testing, NanoMEDIC-Luc containing FRB-fused luciferase protein was injected into the gastrocnemius muscle of mice. Luciferase expression was observed in a dose-dependent manner 16 h post injection, while off target effects were not detected in other organs such as the liver. Luciferase was cleared from the system within 3 days.

Transgenic mice with a luciferase coding sequence driven by the strong synthetic CAG promoter and interrupted by human dystrophin exon 45 flanked by introns were injected with NanoMEDIC containing sgRNA-DMD1 or sgRNA-DMD23 into the gastrocnemius muscle. Luciferase expression was induced after 3 days post injection and lasted for 160 days, which suggests stable maintenance of exon 45 skipping. Approximately 7% exon skipping efficiency and genomic deletion were confirmed by RT-PCR and deep sequencing analysis, respectively [94]. In conclusion, the NanoMEDIC system seems superior to other approaches in terms of achieving in vivo genome editing and raises new hope for patients suffering from DMD.

The fusion of exosomal proteins with RNA binding proteins can greatly increase the loading efficacy of RNA into EVs. Engineered exosomes were obtained by fusing the tetraspanin CD9, an exosomal surface marker, with *HuR* (human antigen R), an RNA binding protein that interacts with adenylate-uridylate-rich elements (AREs) in the target RNA with high affinity [95]. When three AREs were cloned downstream of the dCas9 stop codon, the loading efficiency of dCas9 mRNA into CD9-HuR exosomes was increased 9.3-fold. Interestingly, the addition of three AREs decreased the dCas9 mRNA expression in cells, but increased its encapsulation in exosomes. Exosomes secreted by HEK293T cells expressing sgRNA and dCas9-ARE significantly reduced target gene expression in recipient adipogenic stem cells, while exosomes produced by cells expressing unmodified dCas9 were ineffective. Inhibition was also observed in the murine liver after tail vein injection [95].

Another example of applying EVs for CRISPR/Cas9 delivery is GEDEX, which stands for “genome editing with designed extracellular vesicles” [91]. HEK293 cells were transfected with plasmids to provide overexpression of Cas9 and sgRNA. EVs secreted by these producer cells contained Cas9 and sgRNA, which was confirmed to be encapsulated, not surface-bound, by RNase treatment. GEDEX targeting the MYD88 gene induced DNA double-stranded breaks in HEK293 target cells slightly less efficiently than a plasmid vector carrying *MYD88* (MYD88 innate immune signal transduction adaptor) sgRNA and Cas9. In order to track EVs in vivo, GEDEX targeting the Prostaglandin-Endoperoxide Synthase1 gene (*Ptgs1*), which is involved in angiogenesis and cancer, were labelled with the fluorescent dye DiD (1,1-Dioctadecyl-3,3,3,3-tetramethylindodicarbocyanine) and injected into mice. Fluorescence imagining showed the distribution of GEDEX throughout the organism within 3 h after injection. The T7E1 assay detected indel formation in collected organs, including kidney, brain, spleen, heart, lungs, and liver.

The targeting efficiency of GFP-directed GEDEX was examined in in vitro and in vivo models. Firstly, HEK293-GFP cells were treated with GFP-GEDEX, which resulted in decreased fluorescence in more than 70% cells. Next, GFP-GEDEX were injected intraperitoneally to B6-EGFP mice, which expresses GFP in all cells and tissues. Peritoneal cells were harvested after 5 days. Flow cytometry analysis detected a 50% decrease in eGFP fluorescence.

GEDEX was also successfully used for CRISPR-mediated transcriptional activation. CRISPRa was obtained by fusing dCas9 to the tripartite activator domain VP64-p65-Rta (VPR). HEK293 cells transfected with a firefly luciferase reporter vector and treated with GEDEX containing sgRNA and dCas9-VPR protein showed a significant increase in luciferase activity. Gene upregulation was not impeded by pre-treating GEDEX with proteinase K. On the other hand, a significant decrease in luciferase upregulation was caused by lysing GEDEX with Triton X-100, which shows the protective role of the EV membrane for the cargo. Mice were subcutaneously implanted with HEK293 cells transfected with luciferase reporter and injected with GEDEX one hour later. The bioluminescence caused by upregulation of the luciferase was observed. The ability to upregulate the expression of an endogenous gene was studied with GEDEX carrying dCas9-VPR and a sgRNA targeting the promoter region of the human or mouse *Actc1* (Actin Alpha Cardiac Muscle 1) gene. Seven-fold and five-fold increases of ACTC1 mRNA were observed in human HEK293 cells and mouse Neuro2A cells, respectively.

A promising therapeutic application of the GEDEX system is liver regeneration, which may be achieved by the upregulation of hepatocyte growth factor (HGF) gene expression. Hepatotoxicity was induced in mice models by oral administration of ANIT (alpha-naphthylisothiocyanate). Then, GEDEX targeting HGF were delivered hydro-dynamically. HGF levels in collected livers were increased significantly. The liver damage markers including ALT (Alanin-Aminotransferase) enzyme, bile acids, total bilirubin, and cholesterol were higher in control mice compared to the HGF-GEDEX treated ones. The histological analysis of livers showed significantly less tissue damage, which confirms the therapeutic effect of HGF in liver regeneration. Control animals with ANIT-induced hepatotoxicity suffered from far greater cell infiltration, hyperaemia, and necrosis development. Therefore, the upregulation of genes involved in tissue regeneration is a potential clinical application of CRISPR/Cas9 system delivered by EVs [91].

Luo et al. [92] tried to use exosomes carrying CRISPR/Cas9 components for anti-fibrotic treatment of the murine liver. They used an inactivated variant of Cas9 (dCas9) fused to the VP64 transactivator domain for target site specific activation of gene expression. The genes for the dCas9-VP64 fusion protein and the gRNA directed to the hepatocyte nuclear factor 4a (*HNF4a*) were transfected into the murine producer cells in the form of plasmid DNA. Exosomes from producer cells were isolated and incubated with mouse hepatic stellate cells that are associated with fibrosis. Induced expression of HNF4a caused a phenotypic change of fibrosis associated hepatic stellate cells into hepatocyte-like cells [92].

Another approach of generating extracellular vesicles is the production of so-called “gesicles” [96]; these are microvesicles that are induced by the overexpression of the vesicular stomatitis virus G glycoprotein (VSV-G) during production [104]. Gesicles were used to deliver CRISPR/Cas9 targeting the HIV long terminal repeat regions. HEK293 cells were transfected with mix of plasmids expressing one of four components: VSV-G, CherryPicker Red, *Cas9*, and guide RNA. VSV-G enables gesicle production through membrane fusion. CherryPicker Red is a membrane-associated protein that was expressed as fusion protein with the DmrA domain, which physically interacts with DmrC domain fused with the Cas9 RNP. This interaction is inducible by the addition of the A/C heterodimerizer molecule, which facilitates Cas9 RNP packaging into the gesicle. Cas9 dissociation from CherryPicker Red appears as a result of dilution of the A/C heterodimerizer after gesicle fusion with the cell membrane. Gesicles isolated from HEK293FT cells contained CherryPicker Red, VSV-G, and Cas9, which was confirmed by Western blot analysis. The presence of A/C heterodimerizer predictably increased Cas9 levels in gesicles isolated from HEK293FT cells.

In addition to its association with the Cas9 RNP, CherryPicker Red was used as a fluorescent marker, which permitted the analysis of gesicles as fluorescent particles ranging from ~50 to 100 nm in diameter.

Gesicles were added to human microglia cells (CHME-5) to deliver CherryPickerRed and Cas9. Cells were collected 1 h, 4 h, and 24 h post treatment. CherryPicker Red, VSV-G and Cas9 proteins were detected in CHME-5 cell as soon as 1 h after delivery. Intriguingly, while CherryPicker Red and VSV-G proteins were present for at least 24 h, Cas9 protein was not, which suggests its shorter half-life.

A microglial cell line, HIV-NanoLuc CHME-5, was used to examine the CRISPR/Cas9 delivery by gesicles. HIV-NanoLuc contains a modified HIV provirus with a Nano-Luciferase reporter construct under control of the HIV LTR and produces Nef, which is a HIV protein. The number of HIV provirus and Nef virus copies decreased after gesicle treatment, as was measured by droplet digital PCR. The T7E1 assay confirmed mutations in the targeted LTR region. Moreover, sequencing followed by “tracking indels by decomposition” (TIDE) analysis measured the average of mutation of the 5′-LTR amplified region, which was 8%, while the off-target events efficiency did not exceed the 2% background threshold.

Comparison of gesicle delivery to lipofectamine-mediated transfection of plasmids expressing Cas9 and gRNAs showed that two transfection cycles were needed to decrease the proviral copy number, contrarily to gesicle treatment [96].

Active enrichment of RNP in EVs is an appealing approach for efficient CRISPR/Cas9 genome editing. The specific interaction between an aptamer (“com” as the RNA fragment) and the aptamer-binding protein (ABP) was exploited for the improved loading of RNPs into delivery vectors [97]. Interactions of ABP-“Com” (the protein binding to the com RNA aptamer) fused to CD63, an EVs-specific tetraspanin, and com-modified sgRNA enabled active recruitment of RNPs to EVs. Moreover, the VSV-G protein was included to facilitate endosomal escape after transfer by the vesicles.

HEK293T cells were transfected with three plasmids expressing: ABP-Com fused with CD63, *VSV-G*, and gene editing SaCas9/SpCas9/adenine base editor and com-modified sgRNA. Secreted extracellular vesicles were isolated from cell culture supernatants by ultracentrifugation and added to HEK293T-HBB-IL2RG-EGFP reporter cells. In these cells, EGFP expression is impaired due to the insertion of mutated human *HBB* (haemoglobin beta) and human *IL2RG* (Interleukin 2 Receptor Subunit Gamma) sequences after the EGFP start codon. SaCas9 RNPs delivered by EVs restored EGFP expression by generating indels in the targeted *IL2RG* sequence. The aptamer com modification of the *IL2RG*-targeting sgRNA was crucial for effective gene editing by both SaCas9 and SpCas9 RNPs. Depending on the fusion of the ABP-Com to either N-terminus or C-terminus or both termini of CD63, different outcomes were obtained. The Com-CD63-Com variant resulted in the highest rate of edited cells (~25% for SaCas9 RNP and ~10% for SpCas9), which is calculated by GFP expression that was analyzed by flow cytometry. Fusing Com to the C-terminus of *VSV-G* decreased its expression by half. Moreover, RNP enriched EVs produced by cells in the absence of VSV-G were not functional. Efficient indel formation and adenine base editing were confirmed in multiple targets, including *DMD* (Dystrophin), *GAPDH* (Glyceraldehyde-3-phosphate dehydrogenase), and *P53* in EGFP reporter cells, HEK293T, and MDA-MB-231 cells.

Nanoparticle tracking analysis revealed that Com-CD63-Com and Cas9 RNPs slightly decreased the number of secreted EVs and changed the size distribution by increasing the pool of EVs with a diameter of ~200 nm. One of the features of RNPs delivery is its shorter half-life compared to prolonged expression of Cas9 by viral vectors. The half-life of SpCas9 delivered by EVs to HEK293T cells was approximately 3 h.

A significant advantage of RNP-enriched EVs is their ability to target multiple sites simultaneously. Multiplex gene editing was applied to target intron 50 and 51 of DMD using SaCas9 RNPs targeting both sites. The 2 kb sequence between both introns was successfully removed. Moreover, RNPs targeting *DMD* exon 53 injected into the TA muscle of del52hDMD/mdx mice led to indel formation in vivo [97].

## 9. Direct Exosome Engineering after Their Isolation

In addition, utilizing the cellular packaging machinery for inserting cargo into exosomes, there is another method based on loading isolated EVs with RNA and proteins (Figure 3B).

Kim et al. [98] isolated exosomes from HEK293 (HEK293-Exo) and SKOV3 (SKOV3-Exo) cells. The size range varied from 50 to 150 nm, measured by dynamic light scattering, and a round-shape was observed on TEM (Transmission electron microscopy). Neither the epithelial cell-derived HEK293-Exo nor tumor-derived SKOV3-Exo induced an immune response in PBMCs evaluated by TNF-α (Tumor necrosis factor alpha) and INF- α (Interferon alpha) production. Interestingly, Lipofectamine 2000, a commonly used transfection reagent, was immunogenic, which highlights that exosomes are more suitable than cationic liposomes for in vivo applications.

To further study their functionality, tumor-derived exosomes were packaged with a sgRNA/Cas9 expressing plasmid targeting *PARP-1* (Poly(ADP-Ribose)-Polymerase 1) by using electroporation. *PARP-1* inhibition induced by CRISPR/Cas9 in SKOV3 cells was comparable using cancer-derived exosomes or Lipofectamine 2000. SKOV3-Exo were able to induce indel mutations in *PARP-1* with a 27% efficiency calculated by the T7E1 assay. Moreover, the reduction in tumor volume and weight of SKOV3 xenografts in mice were observed after SKOV3-Exo intravenous or intratumoral injections.

Interestingly, the combined treatment with cisplatin and CRISPR/Cas9 loaded SKOV3-Exo provided a synergistic effect on inhibiting the proliferation of SKOV3 cells to 57% in vitro, while the efficacy of cisplatin and SKOV3-Exo alone were 21.6% and 30%, respectively. Similarly, the cisplatin and iPARP-1/SKOV3-Exo combination showed a higher apoptotic rate of 27.56% compared to 11.06% or 11.9% for cisplatin or iPARP-1/SKOV3-Exo alone, respectively.

FACS analysis showed that the uptake of tumor-derived SKOV3-Exo was higher than the uptake of epithelial cell-derived HEK293-Exo (79% and 67%, respectively) in SKOV3 cells; therefore, cancer-derived exosomes were preferably uptaken by cancer cells compared to epithelial cell-derived exosomes. This pattern was additionally observed in an in vivo experiment, where SKOV3 xenograft mice received intravenous injections of labeled exosomes into tail veins. While SKOV3-Exo accumulated at the tumor sites, HEK293-Exo were also found in other tissues including the liver, which is a common off-target site of accumulation [98].

In a combination of producer cell based and direct exosome engineering, a hybrid form of liposomes and exosomes was used for efficient plasmid DNA transfection of human mesenchymal stem cells (MSCs) [101]. In comparison with the hybrids, lipofectamine or exosomes as delivery vehicles were insufficient; this may be due to the ineffective encapsulation of plasmids by electroporation. Hybrid exosomes were obtained by mixing liposomes, exosomes isolated from HEK293FT cells, and plasmids expressing enhanced green fluorescence protein (EGFP). After incubating the mixture for 12 h at 37 °C, the exosomes fused with liposomes and encapsulated the plasmids.

In order to determine successful plasmid encapsulation, MSCs were incubated with hybrid exosomes in the presence of DNase. The levels of EGFP mRNA in MSCs measured by qRT-PCR did not significantly differ with or without DNase treatment, which indicates that hybrid exosomes protected plasmid DNA from nuclease digestion by fully encapsulating it inside the vesicles. The cellular uptake was probably facilitated by vesicle surface proteins since treatment of proteinase K inhibited EGFP delivery to MSCs. Cell toxicity of hybrid nanoparticles was comparable to lipofectamine, whereas endogenous exosomes did not affect cell viability.

HEK293FT cells were transfected with a vector expressing sgRNA targeting *Runx2* (Runt related transcription factor 2). Exosomes isolated from sgRNA expressing cells were incubated with liposomes and *dCas9* vector for 12 h. The mixture was then added to MSCs. Increased mRNA levels of sgRNA and dCas9 were detected in MSCs, while the level of Runx2 was significantly decreased. CRISPR/Cas9 system delivered by exosomes efficiently inhibited Runx2 expression. The exosomes isolated from the treated MSCs cells contained sgRNA and dCas9 mRNA, as well as dCas9 proteins [101].

Surface modification of extracellular vesicles can improve their functionality for CRISPR applications. Zhuang et al. [99] designed a delivery system composed of DNA aptamers displayed on exosomes that served as vehicles for ribonucleoprotein (RNP) complexes with gene editing activity. RNPs consisting of Cas9 protein and sgRNA were loaded into EVs by sonication or three cycles of freezing and thawing. The latter method turned out to result in more efficient loading (37.62% vs. 15.34%). Then, RNP-loaded EVs were decorated with three-dimensional tetrahedral DNA nanostructures (TDNs). The surface modification did not change the size of the EV (ranging from 100 to 150 nm), however, the zeta potential decreased due to the negative charge of TDNs. The TDNs were used to display the conjugated DNA aptamers on the EV membrane, which enhanced cellular uptake through target cell specific delivery. First, the cholesterol/aptamer ratio of TDN-EVs was optimized to obtain the highest delivery efficacy in HepG2 cells. Then, TDN1-EVs-RNP nanoparticles targeting GFP were added to GFP expressing HepG2 cells. Successful CRISPR mediated indel mutations were confirmed by T7E1 assay, sequencing, and fluorescence reduction in ~43% of cells.

WNT10B (Wnt Family Member 10B) overexpression has been a target for hepatocellular cancer therapy. RNP complexes targeting *WNT10B* were designed to knock it down in HepG2 cells and human primary liver cancer-derived organoids. Application of a TDN-based targeting strategy resulted in a significant reduction in WNT10B protein expression and reduced HepG2 cell viability. In contrast, unmodified EV-RNPs provided a weaker reduction WNT10B protein level and indel formation, while liposomes-RNP did not affect WNT10B significantly. In agreement with these cell culture experiments, TDN1-EV-RNPs inhibited the growth of ex vivo tumor organoids most effectively. Finally, the vesicles were tested in vivo. HepG2 xenograft tumors in mice were examined after intravenous treatment with the modified EVs. Tumor development was abolished by the higher dose (1.0 mg/kg) of TDN1-EV-RNPs. In comparison to unmodified EV-RNPs, TDN1-EV-RNPs at a lower dose (0.1 mg/kg) inhibited tumor growth to a higher degree. Lipo-RNPs did not affect tumor development significantly. Compared to EV-RNPs, TDN1-EV-RNPs caused indel formation (5-fold greater for high doses or 2-fold greater for low doses) at a higher frequency in tumors from xenograft mice [99].

One of the hurdles in studying and applying extracellular vesicles is the difficulty of producing and isolating them [81]. Isolation and purification processes of EVs are time consuming and hardly scalable. The number of cells required to secrete enough EVs is very high, which is difficult to achieve with primary cell culture. Immortalized cell lines are easier to culture, however, there is a risk of transferring oncogenic genetic material. The hazardous horizontal gene transfer can occur in all nucleated cells. An interesting solution to these limitations is to use human red blood cells (RBCs) to produce EVs [100]. RBCs are easily collected from blood in large quantities since they are the most abundant cell type in the human body. Moreover, RBCs do not contain nuclear or mitochondrial DNA which eliminates the risk of horizontal gene transfer.

Group O-RBCs were isolated from blood using centrifugation and leukodepletion filters and treated with a calcium ionophore to stimulate EV release. Sequential centrifugations resulted in the isolation of RBCEVs with a diameter of ~140 nm that were enriched in exosome markers (ALIX and TSG101 - Tumor susceptibility 101) and RBC proteins (Hemoglobin A and Stomatin). The delivery of RBCEVs was studied in the leukemia cell line MOLM13. After 24 h of incubation with fluorescence-labelled RBCEVs, ~99% of MOLM13 cells became fluorescent. The uptake was reduced by 60–70% in the presence of heparin, which suggests that it depended on heparan sulfate proteoglycans.

In order to study the application of RBCEVs in the CRISPR system, extracellular vesicles were electroporated with HA-tagged Cas9 mRNA and added to MOLM13 cells. A portion of the 18% of the loaded Cas9 mRNA were protected from RNase degradation. MOLM13 cells were incubated with electroporated RBCEVs packaged with Cas9 mRNA for 24 h. Cas9 mRNA and Cas9 protein were detected in MOLM13 cells, while unelectroporated Cas9 mRNA was not able to transfect the cells. A guide RNA targeting the human *mir125b-2* locus was used to study the functionality of gene editing using the RBCEVs system. RBCEVs packaged with Cas9 mRNA and miR125b-gRNA were added to MOLM13, resulting in a decrease in miR-125b expression by ~98% and a decrease in miR-125a expression by 90%. Moreover, the expression of BAK1 (BCL2 antagonist/killer 1), which is a direct target of miR-125b increased 3-fold.

293T-eGFP cells were incubated for a week with RBCEVs electroporated with two plasmids expressing Cas9 and GFP gRNA. However, the efficiency of EGFP knockout was only 10%; this is likely due to the size of plasmid DNA. Treating NOMO1-eGFP cells with RBCEVs loaded with Cas9 mRNA and anti-eGFP gRNA in a 6:50 molar ratio resulted in the loss of eGFP in ~32% cells, which indicates that RBCEVs provide more efficient delivery of RNA compared to larger plasmid DNA [100].

MYC overexpression occurs in 30% of human cancers, including Burtkitt’s lymphoma, diffuse large cell lymphoma, multiple myeloma, and acute lymphocytic leukemia [105]. Therefore, targeting MYC using CRISPR/Cas9 system may lead to a promising clinical outcome in cancer therapy. Employing the chimeric-antigen receptor (CAR) can improve selective targeting of tumors [102]. An anti-CD19-CAR-HEK293T cell line was generated from HEK293T cells by transfection with a pTRPE lentiviral vector encoding a CAR containing an anti-CD19 single-chain variable fragment. Anti-CD19-CAR-EVs and unmodified EVs were isolated from transfected and control HEK293T cells and subsequently electroporated with MYC-targeting sgRNA/Cas9 plasmids. The CD19^+^ Burkitt’s lymphoma cell line Raji, CD19^+^ acute lymphoblastic leukemia cell line Nalm6, and CD19^−^ chronic myelogenous leukemia cell line K562 were incubated with loaded anti-CD19-CAR-EVs or unmodified EVs. Interestingly, cell proliferation was inhibited by both CRISPR-loaded unmodified and modified EVs, which suggests that extracellular vesicles are effective for plasmid delivery. However, the proliferation of CD19^+^ Raji and Nalm6 cells was stronger decreased when treated with anti-CD19-CAR-EVs compared to unmodified EVs. In case of CD19^−^ K562 cells, no difference in proliferation was observed after incubation with modified or unmodified EVs. Moreover, increased apoptosis was induced by CRISPR-loaded anti-CD19-CAR-EVs (33.8%) compared to loaded unmodified EVs (13.69%) in Raji cells. Therefore, decreased cell viability was more pronounced in CD19^+^ cells through increased antigen-receptor tropism-facilitated targeting of EVs. The efficiency of *MYC*-targeting sgRNA/Cas9 GFP tagged plasmid transfection was measured by tracking GFP expression in Raji cells. The GFP levels were significantly higher (7.38%) after 48 h of incubation with anti-CD19-CAR-EVs compared to unmodified EVs (3.75%). Indel formation with a frequency of 5.71% in Raji cells was confirmed by Sanger sequencing.

The biodistribution of Cy5.5 labelled EVs or anti-CD19-CAR-EVs was then studied in vivo in Raji xenografts in mice. Intracardially injected anti-CD19-CAR-EVs accumulated mostly in targeted tumor sites, while unmodified EVs were found also in other tissues. The portion of fluorescent Raji tumor cells penetrated by Cy5.5 labelled anti-CD19-CAR-EVs were higher (23.9%) as compared to labelled unmodified EVs (13.8%). In order to test the functionality, mice with established Raji subcutaneous xenografts models were intracardially or intratumorally injected five times every fourth day with unmodified/modified MYC-targeting sgRNA/Cas9 loaded EVs as well as EVs only, *MYC*-targeting sgRNA/Cas9 plasmids, and modified EVs loaded with only Cas9. A significantly decreased tumor volume was observed only after the injection of *MYC*-targeting sgRNA/Cas9-loaded anti-CD19-CAR-EVs. Moreover, reduction in the percentage of proliferating Ki67-positive tumor cells, decreased MYC expression, and induced apoptosis also occurred in the tumors treated with loaded modified EVs. Indel formation at the target site with a frequency of 1.4–1.8% was confirmed by Sanger sequencing. The relatively low frequency may be caused by apoptosis of cells after inhibiting MYC, which regulates cell proliferation and so the surviving cells may mostly be those with unedited genes [102].

He et al. [106] employed epithelial cell derived microvesicles (MVs) to transport CRISPR/Cas9 components into tumor cells. The MVs were isolated from HEK293 cells stably transfected with Cas9 and loaded with plasmids encoding for sgRNAs targeting the IQ-domain GTPase-activating proteins. The expression of these proteins is altered in cancer and they are considered as veritable drug targets. Uptake of the loaded MVs reduced the viability of HepG2 liver cancer cells in vitro and a synergistic effect was demonstrated for combinations with sorafenib, which is a multi-kinase inhibitor. A substantial anti-cancer effect was also observed in vivo in a HepG2 xenograft mouse model [106].

## 10. Conclusions

The scientific advancement that was initiated by the successful application of the CRISPR/Cas mechanism for editing the genome of various organisms will continue during the coming years. Starting from a single tool enabling site-directed cut in the genome, we witnessed the rapid development of the assortment of instruments for the highly specific activation or inhibition of gene expression or the exchange of single bases. Modifications at specific genomic loci are not restricted to the DNA itself and, also, localized histones can be targeted and modified. The first clinical applications of the CRISPR/Cas technology were initiated and their results are promising. Several problems associated with the technology still have to be conquered, including off-target effects and safe and efficient delivery. A perfect vehicle for delivery is not only non-toxic but also targets the desired cells, is not immunogenic, is stable for the optimal application time, is highly efficient, and easy to produce in sufficient amounts. In addition to a huge variety of synthetic carriers for CRISPR/Cas components, extracellular vesicles were successfully employed for that task. EVs as natural parts of living organisms bring along many advantageous features for the transport of nucleic acids and ribonucleoproteins into the right intracellular compartment in vivo. Therefore, we think that the future perspectives are very promising for the usage of EVs to deliver CRISPR/Cas molecules into target cells for therapeutic purposes.

## Figures and Tables

**Table 1:** Comparison of the various EV production methods used for transfer of CRISPR/Cas9 components.

	EV Production Method	Advantages	Disadvantages	References
**Producer cell-based exosome engineering**	Transfection of producer cells with RNAs and/or plasmids.	Rapid and simple; universally applicable; unlimited targeting options.	Low (sometimes zero) gene editing efficiency.	[86,90,91,92]
	Active enrichment of Cas9 protein and sgRNAs in vesicles.	Highly active in gene editing; versatile; quantitative measurements of transfer activity (silencing and uptake).	More time and effort required to establish the system.	[93,94,95,96,97]
**Engineering of isolated exosomes**	Loading of RNA and/or protein into purified sEVs.	Better control of cargo loading; for in vivo applications EVs can be isolated from bodily fluids.	Difficulties in obtaining large quantities of EVs; additional EV purification steps required.	[98,99,100]
**Combination of producer cell and EV manipulation**	Isolation of EVs from engineered cells, followed by cargo loading.	Combining advantages from two systems, e.g., targeting provided by engineered cells and efficient loading in vesicles.	Challenging to establish the system; higher number of EV purification steps.	[101,102]

## Data Availability

Not applicable.
